# A motif-based search in bacterial genomes identifies the ortholog of the small RNA Yfr1 in all lineages of cyanobacteria

**DOI:** 10.1186/1471-2164-8-375

**Published:** 2007-10-17

**Authors:** Björn Voß, Gregor Gierga, Ilka M Axmann, Wolfgang R Hess

**Affiliations:** 1 University of Freiburg, Faculty of Biology, Experimental Bioinformatics, Schänzlestr. 1, D-79104 Freiburg, Germany; 2 Humboldt University Berlin, Institute for Theoretical Biology, Invalidenstrasse 43, D-10115 Berlin, Germany

## Abstract

**Background:**

Non-coding RNAs (ncRNA) are regulators of gene expression in all domains of life. They control growth and differentiation, virulence, motility and various stress responses. The identification of ncRNAs can be a tedious process due to the heterogeneous nature of this molecule class and the missing sequence similarity of orthologs, even among closely related species. The small ncRNA Yfr1 has previously been found in the *Prochlorococcus/Synechococcus *group of marine cyanobacteria.

**Results:**

Here we show that screening available genome sequences based on an RNA motif and followed by experimental analysis works successfully in detecting this RNA in all lineages of cyanobacteria. Yfr1 is an abundant ncRNA between 54 and 69 nt in size that is ubiquitous for cyanobacteria except for two low light-adapted strains of *Prochlorococcus*, MIT 9211 and SS120, in which it must have been lost secondarily. Yfr1 consists of two predicted stem-loop elements separated by an unpaired sequence of 16–20 nucleotides containing the ultraconserved undecanucleotide 5'-ACUCCUCACAC-3'.

**Conclusion:**

Starting with an ncRNA previously found in a narrow group of cyanobacteria only, we show here the highly specific and sensitive identification of its homologs within all lineages of cyanobacteria, whereas it was not detected within the genome sequences of *E. coli *and of 7 other eubacteria belonging to the alpha-proteobacteria, chlorobiaceae and spirochaete. The integration of RNA motif prediction into computational pipelines for the detection of ncRNAs in bacteria appears as a promising step to improve the quality of such predictions.

## Background

Non-coding RNAs (ncRNAs) are sequence-specific regulators of gene expression, mediating a plethora of cellular responses to changing environmental conditions and morphological differentiation. In bacteria, ncRNAs are a heterogeneous group of functional RNA molecules normally (but not always) lacking a protein-coding function. They are frequently smaller than 200 nt in size, and act to regulate mRNA translation/decay but can also bind to proteins and thereby modify protein function (recent reviews [1,2]). In many cases, these ncRNAs function through sequence-specific base pairing; hence they frequently have a (partial) base complementarity to their target RNA molecules. The vast majority of known ncRNAs is encoded at genomic locations far away from the target genes (*trans*-encoded ncRNAs). However, a small number of ncRNAs is transcribed from the reverse complementary strand of the respective target and hence these are fully or partially overlapping with their target RNAs. This latter class of ncRNAs is called *cis*-encoded or antisense RNAs.

As a result of recent systematic searches, more than 70 ncRNAs are now known in *E. coli*, most of which had been overlooked by traditional genome analysis. Many, or possibly most major stress responses in *E. coli *include at least one small regulatory RNA as part of the regulon [2]. Systematic biochemical, genetic, genomic or computational searches for ncRNAs are still lacking for most eubacterial phyla outside the enterobacteria. In general, genes encoding ncRNAs are not annotated during standard genome analysis procedures. The efforts to accomplish their identification in bacteria can broadly be divided into (i) sequencing the population of sRNAs (directly or after cloning) as comprehensively as possible (RNomics) or (ii) prediction by bioinformatics tools (mostly) followed by experimental verification (see [3] for a recent review).

There is currently only very scarce information on regulatory RNAs and their genes in cyanobacteria. In addition to the more common types of ncRNA (tmRNA, 6S RNA, RNAse P RNA and *ffs *RNA), less than 10 different ncRNAs and only three antisense RNAs have been described for this whole group of bacteria so far [4-6]. Among the known ncRNAs are Yfr1-7 (cYanobacterial Functional RNA). The existence of these ncRNAs was predicted for one (Yfr4 and Yfr5), two (Yfr3 and Yfr6), three (Yfr1) or four (Yfr2 and Yfr7) strains of the *Prochlorococcus/Synechococcus *lineage in a comparative genomics-based approach and their actual presence was demonstrated under various growth and stress conditions that they encounter in the natural environment [7]. However, functions and phylogenetic distribution of these ncRNAs have remained unknown. There are 19 genome sequences from the *Prochlorococcus/Synechococcus *clade in the public domain now, providing an excellent data set for comparative genomics-based computational prediction of ncRNAs. However, for more distantly related cyanobacteria there are only few such data sets available, rendering the direct identification of ncRNAs through comparative genomics difficult. An exception is ncRNA Yfr7. Due to its exceptionally highly conserved sequence and based on an extended structural model, this RNA was identified as the cyanobacterial homolog of a ubiquitous eubacterial riboregulator, the RNA polymerase – interacting 6S RNA [8]. Molecular details of its expression in synchronized cell cultures of *Prochlorococcus *MED4 are given in more detail separately [9]. The RNAs Yfr2 through Yfr5 in *Prochlorococcus *MED4 constitute a family of closely related ncRNAs with a length of 89–95 nt [7]. Gene copy numbers of Yfr2-5 homologues in other *Prochlorococcus *and *Synechococcus *genomes seem to differ widely and there is only very little synteny among them. Therefore, here we selected Yfr1, with 54, 57 and 56 nucleotides in *Prochlorococcus *MED4, MIT 9313 and *Synechococcus *WH 8102 the smallest ncRNA detected in cyanobacteria so far [7], to address the question if it is possible to find more orthologs of a short bacterial ncRNA in an alternative computational-experimental approach. This approach is likely to be successful also in other cases in which comparative data suggest a highly conserved ncRNA secondary structure that can be taken as a starting point.

## Results and discussion

### Yfr1 in marine unicellular cyanobacteria

Cyanobacteria are phototrophic bacteria which perform oxygenic photosynthesis and populate widely diverse environments such as freshwater, the oceans, rock surfaces, desert soil or the Polar Regions. Therefore, various types of regulatory RNA can be expected that interplay with the different signal transduction pathways and stress responses. Antisense RNAs found within the gas vesicle operon in *Calothrix *PCC 7601 [4], or covering the *ferric uptake regulator *gene *furA *in *Anabaena *PCC 7120 over its full length [6], or regulating the light-absorbing protein IsiA under conditions of iron limitation and redox stress in the unicellular *Synechocystis *PCC 6803 [5] are three known examples for such RNAs in cyanobacteria. Based on comparative computational analysis, we predicted a whole set of putative trans-acting ncRNAs with unknown function, which we called Yfr1-Yfr7 [7].

**Figure 1:**
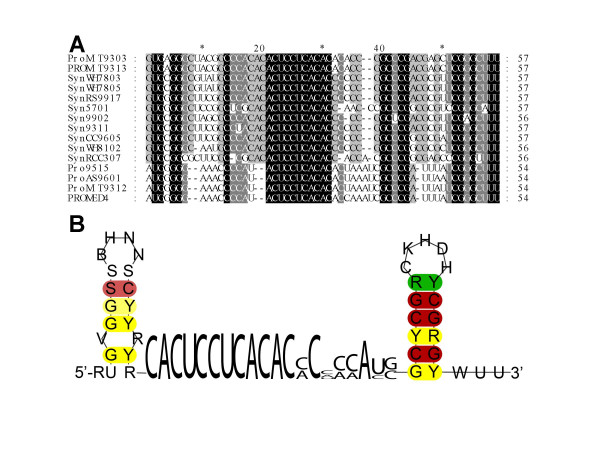
**Comparison of putative Yfr1 RNAs from 15 different *Prochlorococcus *and *Synechococcus***. A. Sequence alignment of the top scoring sequences obtained by BlastN using the known Yfr1 sequences [7] as a query. The respective strain numbers are prefixed "Pro" and "Syn" for *Prochlorococcus *and *Synechococcus*. B. Sequence/structure model for putative Yfr1 RNAs from 15 different *Prochlorococcus *and *Synechococcus *as shown in part A. Sequence is given in IUPAC-notation (R: A or G; Y: C or U; S: G or C; K: G or U; B: G, U or C; V: G, C or A; D: G, U or A; H: A, C or U). Base pair colors indicate the number of different base pairs which occur in the different sequences at this position (red = 1, yellow = 2, green = 3) and their shading resembles the frequency of base pairing, i.e. the number of sequences where this base pair is not present.

We have previously shown the existence of Yfr1 in three out of four tested marine cyanobacteria belonging to the genera *Prochlorococcus *and *Synechococcus *[7]. Unicellular marine cyanobacteria of these genera provide an excellent dataset for computational predictions that require comparative genome information since currently 19 different genome sequences from very closely related isolates are available. Thus, there is an extensive dataset to take compensatory base mutations into account for the prediction of novel ncRNA candidates. In these genomes, orthologs of Yfr1 can be found even by simple BLAST searches (Fig. 1A).

### Prediction of Yfr1 homologs in different classes of cyanobacteria based on an RNA motif

Despite some recent progress [10], marine unicellular cyanobacteria are not trivial in functional studies requiring genetic manipulation. Therefore, finding possible orthologs of Yfr1 in one of the cyanobacterial model species amenable for genetic manipulation would be desirable. The direct identification of such an ncRNA is not intuitively possible based on sequence similarity alone. In Table 1, BlastN hits are given for three such model strains, *Synechocystis *PCC 6803, *Synechococcus *PCC 7942/6301, *Anabaena *PCC 7120 and the marine *Synechococcus *WH 7803. Using the *yfr1 *full length sequence of *Prochlorococcus *MED4 (5'-atgggggaaaccccatactcctcacacaccaaatcgcccgatttatcgggcttt-3') as a query, only for *Synechocystis *PCC 6803 a hit to the correct sequence was found, ranking only at the third position. Similar results were obtained when the *yfr1 *full length sequence of *Synechococcus *WH 8102 was taken, except that the ortholog in the very closely related *Synechococcus *WH 7803 was found with clear statistical support (score = 77.8 bits, e-value = 4e-16; not shown).

**Table 1:** Search for homologs of *Prochlorococcus *MED4 Yfr1 within the genome sequences of four different cyanobacterial model species based on sequence similarity

**Species**	**Match**	**Score (bits)**	**E value**	**Location**
***Synechocystis *PCC 6803**		30.2	0.13	overlapping the start codon of slr1277 *gspD *in antisense orientation
		30.2	0.13	intergenic spacer sll0185_slr0199
		28.2	**0.50**	**ssl3769-*trxA *intergenic spacer**
***Anabaena *PCC 7120**		32.2	0.058	mRNA, cobalt transport protein
		28.2	0.91	mRNA, hypothetical protein
		28.2	0.91	Intergenic spacer
***Synechococcus *PCC 630**1		26.3	1.4	antisense orientation to mRNA of syc1906_d "hypothetical protein"
		26.3	1.4	mRNA,*ftsY*
		24.3	5.4	mRNA, *hisC*
***Synechococcus *WH 7803**		24.3	4.8	mRNA, Ferredoxin-dependent glutamate synthase
		24.3	4.8	mRNA, NAD/NADP transhydrogenase subunit alpha part 1
		22.3	19	mRNA, glycosyltransferase of family GT4

The three top hits (S, subject sequence; Q, query sequence) obtained by BlastN are given for each genome.

**Figure 2:**
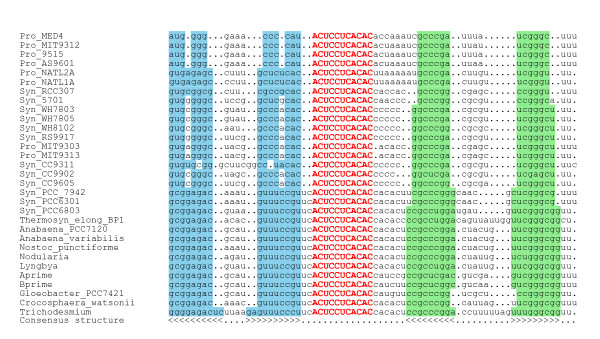
**Multiple alignment of Yfr1 RNA from 31 cyanobacteria**. Blue and green background colors indicate participation in a base pair of the consensus structure, which is given on the last line. The perfectly conserved sequence motif within the unpaired region is given in capital, red letters.

Thus, sequence homology alone is not sufficient and we reasoned to complement it by comparative structure information. Taking up this idea, the initial set of the three previously identified Yfr1 sequences from *Prochlorococcus *MED4, MIT9313 and *Synechococcus *WH8102 plus the novel putative orthologs from the marine *Synechococcus/Prochlorococcus *were subjected to a comparative sequence/structure analysis. As a result we derived an initial sequence/structure-model (Fig. 1B) consisting of two stem loops and a central unpaired section of 18–19 nt. Interestingly, both loop sequences appeared unconserved and therefore these were set to aNy nucleotide in our subsequent searches. In contrast, we noted the presence of an eleven nt highly conserved sequence stretch within the unpaired central section. We reasoned if this sequence element would be essential and tried different RNA motif descriptors in which the undecanucleotide had to be fully conserved or allowed one or two mismatches. Furthermore, we relaxed the required length of the central unpaired section to a range of 12–25 nt and the lengths of both terminal helices to 5–10 base pairs and permitted a single bulge or mismatch in the 5' helix as observed in the data in Fig. 1. This model was used to search for orthologs within genomes of other cyanobacteria and also other eubacteria. Candidates found by this method were incorporated into the model and, finally, this procedure led to the identification of putative Yfr1 orthologs in 31 (28 new) out of 33 available cyanobacterial genomes. All here identified Yfr1 candidates are summarized in a multiple sequence/structure alignment shown in Fig. 2. An outstanding property appearing in this alignment is the perfectly conserved 11 nt long sequence motif (5'-ACUCCUCACAC-3') within the unpaired region. The reason for this perfect conservation might be that this short sequence is targeting a conserved sequence element in one or several other RNAs, mRNAs or ncRNAs, through base-pairing interactions as it is known for many ncRNAs. Alternatively, it could also be a protein binding motif. The rather high concentration of Yfr1 in the different organisms is rather untypical for an ncRNA acting through base-pairing.

**Figure 3:**
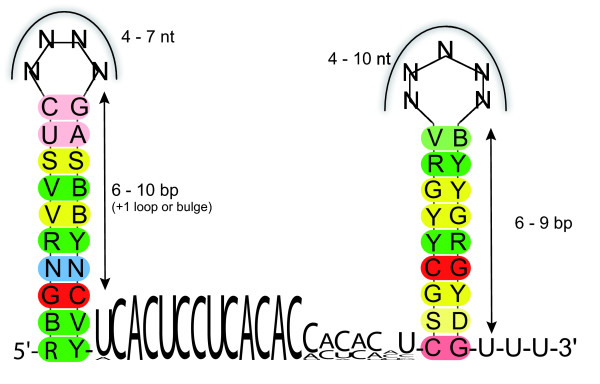
**Sequence/structure model for Yfr1 RNA based on a structural alignment created from a ClustalW multiple sequence alignment and an RNAlishapes consensus secondary structure from 31 cyanobacteria**. Sequence is given in IUPAC-notation (R: A or G; Y: C or U; M: A or C; S: G or C; B: G, U or C; V: G, C or A; D: G, T or A). Base pair colors indicate the number of different base pairs which occur in the different sequences at this position (red = 1, yellow = 2, green = 3 and blue = 4) and their shading resembles the frequency of base pairing, i.e. the number of sequences where this base pair is not present. The unpaired sequence is given as a sequence logo prepared using WebLogo [23].

From this set of orthologs, a comprehensive sequence/structure-model for Yfr1 was derived (Fig. 3). The 5'-end forms a 6–10 bp stem-loop, which may be interrupted by one bulge or internal loop. At the 3'-end the sequences show a 6–9 bp GC-rich hairpin, followed by a U-tail. These features make this element very likely to be a Rho-independent terminator. Furthermore, together with the 5'-terminal helix the 3'-helix may ensure the unpaired state of the conserved sequence motif and/or protect the RNA against degradation. One would expect approximately 0.5 instances of a specific 11 nt motif in a 2 MB genome and equal base distribution of 0.25. The here identified ultraconserved 5'-ACUCCUCACAC-3' occurs more often then by chance in the investigated genomes (for instance, three times in *Prochlorococcus *sp. MED4, genome size 1.66 MB; four times in *Synechocystis *PCC6803, genome size 3.57 MB, but also twice in *Prochlorococcus *sp. SS120 where it is not related to Yfr1 and is not located within a comparable context of possible secondary structure). Therefore, this motif alone is not sufficient to find homologs of Yfr1. However, it is interesting to note that the two additional occurrences in *Prochlorococcus *MED4 both are located in intergenic spacer regions, close to two other ncRNAs: 160–150 nt upstream the mapped 5' end of Yfr2 and 128–138 nt upstream of Yfr5 [7]. The only two cyanobacterial genomes for which no Yfr1 ortholog was found were *Prochlorococcus *sp. SS120 and MIT9211. These latter two examples were chosen to test when the RNA motif prediction would find likely false positives. When the score was reduced to 0.0 still no candidate Yfr1 homologs were found in these two genomes. Only when in addition one or two mismatches were allowed in the central consensus element, candidates were found (see additional file 1). However, these candidate sequences could not be aligned to each other or to the other Yfr1 sequences. In case of *Prochlorococcus *sp. SS120 also a previous experimental screen had remained negative [7]. Therefore, the candidates found under these very relaxed conditions (additional file 1) do not appear realistic. Thus, the here desribed search method allows for an  excellent discrimination between true positives and false  positives. This finding is in agreement with studies in which RNA motif prediction was integrated into computational pipelines for the high-throughput prediction of *cis*-regulatory RNA sequences [11].

Conservation of genomic location and flanking genes can also be a powerful tool for finding related ncRNAs. Indeed, the *yfr1 *gene is at a conserved position in the majority of the here investigated genomes. In 25 out of 31 genomes, it is found upstream a *guaB *gene, coding for inosine-5'-monophosphate dehydrogenase (Table 2). Even more obvious is the frequent association with a downstream located *trxA *gene (27 of 31 genomes, Table 2).

**Table 2:** Genomic location and orientation of *yfr1 *in cyanobacteria

**Organism**	**Orientation**		
all *Prochlorococcus *(8 strains)	*guaB*→	***yfr1*→ **	*trxA*→
all marine *Synechococcus *(9 strains)	*guaB*→	***yfr1*→ **	*trxA*→
*Thermosyn. elongatus*	tlr1779*→	***yfr1*→ **	←tll1780*
*Gloeobacter violaceu*s	*trpE*→	***yfr1*→ **	*trxA*→
*Synechococcus *Bprime	*ligA*→	***yfr1*→ **	*clpX*→
*Synechococcus *Aprime	*ligA*→	***yfr1*→ **	*clpX*→
*Synechococcus *PCC7942/6301	*guaB*→	***yfr1*→ **	*trxA*→
*Crocosphaera watsonii *WH 8501	←*pcrA*	***yfr1*→ **	*trxA*→
*Synechocystis *PCC 6803	←sll0586*	***yfr1*→ **	*trxA*→
*Trichodesmium eryt*. IMS101	*guaB*→	***yfr1*→ **	←Tery3307*
*Lyngbya *PCC7419	*guaB*→	***yfr1*→ **	*trxA*→
*Anabaena variabilis *ATCC 29413	*guaB*→	***yfr1*→ **	*trxA*→
*Anabaena *PCC7120	*guaB*→	***yfr1*→ **	*trxA*→
*Nostoc punctiforme *PCC73102	*guaB*→	***yfr1*→ **	*trxA*→
*Nodularia *PCC9350	*guaB*→	***yfr1*→ **	*trxA*→

See Fig. 2 for the strain names of marine *Synechococcus *and Prochlorococcus;*hypothetical protein.

We extended the search also to non-cyanobacterial photosynthetic bacteria, such as the alpha-proteobacteria *Erythrobacter litoralis*, *Rhodobacter sphaeroides*, *Silicibacter sp*. TM1040, the chlorobiaceae *Chlorobium tepidum*, *Chlorobium chlorochromatii *and *Cytophaga hutchinsonii*, as well as one spirochaete (*Leptospira borgpeterseni*) and, finally, *Escherichia coli*, but did not identify a reasonable ortholog of Yfr1 within these. So far, the occurrence of Yfr1 seems to be restricted to cyanobacteria. Since cyanobacteria represent a separate eubacterial phylum for at least 2.5 billion years of evolution it is very likely that Yfr1 originated very early in the progenitors of this group and must have co-evolved with its (hypothetical) target(s) for a very long time.

### Experimental verification of Yfr1 in phylogenetically different groups of cyanobacteria

**Figure 4:**
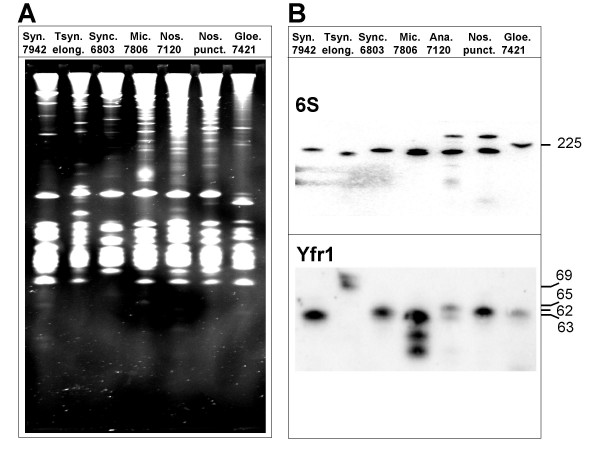
**Detection of Yfr1 RNA in seven different freshwater cyanobacteria**. **A**. About 10 μg of total RNA from *Synechococcus *sp. PCC 7942 (Syn 7942), *Thermosynechococcus elongatus *BP1 (Tsyn. elong.), *Synechocystis *PCC 6803 (Sync. 6803), *Microcystis aeruginosa *PCC 7806 (Mic. 7806), *Nostoc *sp. PCC 7120 (Nos. 7120), *Nostoc punctiforme *(Nos. punct.) and *Gloeobacter violaceus *PCC 7421 (Gloe. 7421) was analyzed by staining a 10% polyacrylamide gel with ethidium bromide.**B**. Northern blot hybridization with DNA oligonucleotides for the presence of Yfr1 (lower part) and, as a control, the 6S RNA (upper part).

According to morphologic criteria, cyanobacteria can be organized into five different sections [12]. These sections are only partially congruent with molecular phylogenetic data, therefore we chose six cyanobacterial species which clearly are very different from each other, judged by their morphology, 16S rRNA sequence and life style, to test our result by Northern hybridization. We also included RNA from *Microcystis *PCC7806 for which no genome sequence was available and, hence, no candidate predicted. The results are given in Fig. 4 and clearly show that Yfr1 is present in all tested species. Together with the three previous identified ones [7] our experiment raises the number of validated Yfr1 homologs to ten. In our previous study we did not identify an Yfr1 homolog in *Prochlorococcus *SS120. This is substantiated by the result of this study as we also do not predict one here. Summarizing, we found reasonable orthologs of Yfr1 in 31 out of 33 cyanobacterial strains and, furthermore, their existence could be validated for ten out of ten in this and a previous study.

**Figure 5:**
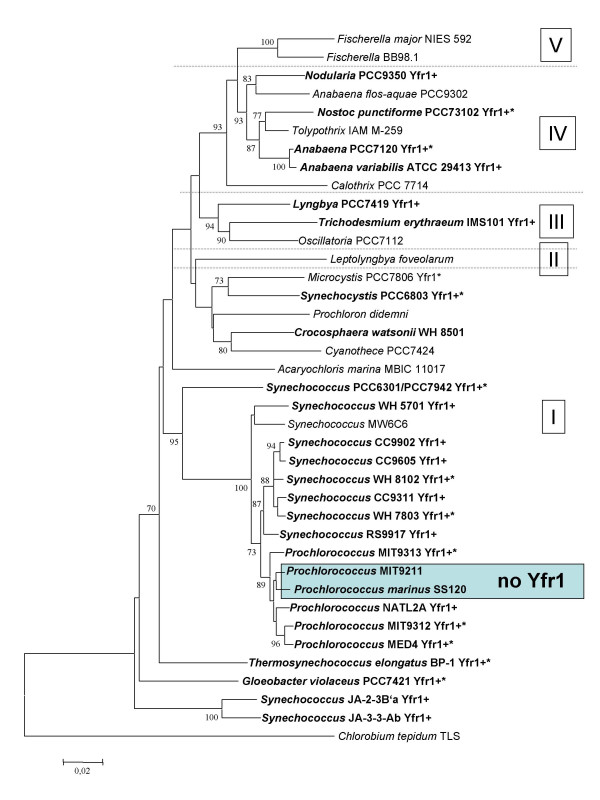
**16S rRNA-based phylogenetic tree summarizing the presence or absence of Yfr1 in all major cyanobacterial lineages**. Cyanobacteria for which genome sequences are currently available from public databases are in boldface letters. Those strains, for which a Yfr1 ncRNA was computationally predicted are labeled Yfr1+. Those, for which we provide experimental evidence for the presence of Yfr1 are labeled Yfr1*. We have shown the presence of Yfr1 in the *Prochlorococcus *strains MED4 and MIT9313 and in *Synechococcus *WH 8102 before [7]. The tree is based on a multiple sequence alignment of 16S rRNA sequences analyzed in MEGA3.1 [24]. Statistic support values were generated by bootstrapping and are only shown if > 70. The 16S rRNA of *Chlorobium tepidum *TLS served as an outgroup. The sections I-V of cyanobacterial taxonomy [12] are indicated.

Superimposing our results with a phylogenetic analysis based on 16S rRNA yields the tree shown in Fig. 5. The widespread existence of Yfr1 over large evolutionary distances points to an important function of Yfr1 throughout the cyanobacterial domain. Interestingly, *Prochlorococcus *SS120 and MIT9211, neither of which is predicted to hold an Yfr1 homolog, appear in one separate cluster. This lets us conclude, that these two or a common ancestor of them lost Yfr1 secondarily. This may be related to their special habitat. Different *Prochlorococcus *isolates can broadly become subdivided in an ecotype adapted to low light and another one adapted to high light. These ecotypes are genetically and physiologically distinct [13] and show a distinct distribution under natural conditions [14,15]. *Prochlorococcus *SS120 and MIT9211 have both been isolated from greater depths in the water column and are adapted to very low light conditions.

## Conclusion

Starting with a known ncRNA from one lineage we show here the highly specific and sensitive identification of its homologs within all lineages of cyanobacteria. The integration of RNA motif prediction into computational pipelines for the detection of ncRNAs in bacteria appears as a promising step to improve the quality of such predictions. The only cyanobacteria that appear to lack Yfr1 are  *Prochlorococcus* SS120 and MIT9211, which both are adapted  to live at very low light intensities and must have lost  Yfr1 secondarily. Therefore, one essential function of Yfr1 might have to do with the ability to cope with high photosynthetic energy fluxes or redox conditions.

## Methods

### Cultures of cyanobacteria

Marine unicellular cyanobacteria were grown as previously published [7]. Cultures of *Microcystis aeruginosa*, *Synechococcus elongatus *PCC 7942, *Thermosynechococcus elongatus *BP-1, *Synechocystis *sp. PCC 6803, *Nostoc *sp. PCC 7120 and *Gloeobacter violaceus *were obtained from the Pasteur Culture Collection and one, *Nostoc punctiforme *ATCC 29133, from American Tissue Culture Collection. All cultures were grown as recommended by the Pasteur Culture Collection.

### Extraction and analysis of RNA

Total RNA was isolated as previously described [7] but with modified lysis conditions for PCC strains, *Anabaena *and *Nostoc *as follows: Disruption by adding an equal volume of glass beads, 33.3 μl 20 % SDS solution and 583 μl acidic phenol to 500 μl concentrated cell solution, following several cycles of vigorous agitation, freezing in liquid nitrogen and thawing in a water bath. Centrifugation of the mixture for 15 min at maximal speed at 4°C yielded an upper aqueous phase which could be cleaned up by standard phenol-chloroform-extraction. Finally, the phases were separated by centrifugation at 9.000 rpm for 15 minutes at 4°C. The RNA was precipitated from the aqueous phase with 100 μl isopropanol for several hours at -20°C, pelleted by centrifugation, washed with 75% ethanol, dried and resuspended in 100 μl of DEPC-treated water. Total RNA was separated in 10 % polyacrylamide-urea gels. Polyacrylamide gels were stained with ethidium bromide (0.3 μg/l) in 1× TBE buffer, rinsed with water and analyzed with a Lumi-Imager F1 system (Roche). Transcript sizes were determined by correlation to *Msp*I-digested DNA of plasmid pUC19.

### Sequence data

The *Nostoc punctiforme *ATCC 29133 (=PCC 73102) genome sequence was downloaded from the JGI website [16]. *Synechococcus *RCC307 and WH7803 were obtained from the Genoscope [17].

All other sequences were obtained from the finished or unfinished genomes website at Genbank [18] with the following accession numbers: *Anabaena variabilis *ATCC 29413, CP000117; *Crocosphaera watsonii *WH8501, AADV00000000; *Gloeobacter violaceus *PCC 7421, BA000045; *Lyngbya aestuarii *CCY9616; *Nodularia spumigena *CCY9414, *Anabaena *PCC 7120, NC_003272; *Prochlorococcus *strains: MIT9312, NC_007577; NATL2A, NC_007335; MIT9313, NC_005071; SS120, NC_005042; MIT9211, AALP00000000; MIT9303, NC_008820; AS9601, NC_008816; NATL1A, NC_008819; MIT9515, NC_008817; MED4 (=CCMP1986), NC_005072; *Synechococcus *strains: PCC7942, NC_007604; PCC 6301, NC_006576; OS-B' (JA-2-3B'a(2-13)), NC_007776; OS-A (JA-3-3Ab), NC_007775; CC9605, NC_007516; CC9902, NC_007513; WH8102, NC_005070; WH7805, AAOK00000000; WH5701, AANO00000000; RS9917 (=RCC556), AANP00000000; RS9916 (=RCC555), NZ_AAUA00000000; *Synechococcus *BL107, NZ_AATZ00000000; *Synechococcus *CC9311, NC_008319; *Synechocystis *sp. PCC 6803, NC_000911; *Thermosynechococcus elongatus *BP-1, NC_004113; *Trichodesmium erythraeum *IMS101, NC_008312.

### Comparative sequence/structure analysis

Multiple sequence alignments were generated using ClustalW [19] with default parameters for DNA. Comparative structure prediction was done with RNAlishapes [20], a tool which predicts a consensus structure for a set of aligned sequences by taking covariance and free energy into account. The resulting consensus structure was analysed together with the multiple sequence alignment using RALEE [21]. The latter served also for manual optimisation of the alignment and the consensus structure, respectively, and for the production of color annotated alignments. Homology searches were done using RNAMotif [22] based on a combined sequence structure motif description for Yfr1. The final RNAMotif descriptor was:

descr

   h5(minlen = 5, maxlen = 10, mispair = 1, pair+={"g:u","u:g"}) ss(minlen = 3, maxlen = 8) h3

   ss(minlen = 12, maxlen = 20, seq="ACTCCTCACAC", mismatch = 0)

   h5(minlen = 6, maxlen = 9, mispair = 0, pair+={"g:u","u:g"}) ss(minlen = 3, maxlen = 15) h3

   ss(seq=" ^ [AUCG]UU$")

score

   { gcnt = 0;

      len = length(h5 [5]);

      loop = length(ss [6]);

      for(i = 1; i < = len; i++){

         j = len - i + 1;

         b1 = h5 [5, i, 1];

      b2 = h3 [7, j, 1];

         if((b1 == "g" && b2 == "c") || (b1 == "c" && b2 == "g"))

         gcnt++;

      }

   # require 65% GC in the stem!

      SCORE = 1.0 * gcnt/len;

      if(SCORE < .65)

      REJECT;

   }

### Oligonucleotides for hybridization

Yfr1_multi_2 5'-GTGTGGTGTGAGGAGTGAACGGAA-3'

Yfr1_gloeo_2 5'-CATGGTGTGAGGAGTGAACGGAAAC-3'

SsA_Thermorev 5'-GCGACGCCGTTTTACCT-3'

SsaA_6803rev 5'-CACCACGCCGTTTTACCT-3'

SsaA_7120rev 5'-CGCAACGCCGTTTTACCT-3'

### Hybridization conditions

Hybridization was performed at 54°C for Yfr1 and 50°C for 6S RNA in hybridization buffer (50% deionized formamide, 7% SDS, 250 mM NaCl, 120 mM Na(PO_4_), pH 7.2).

## Abbreviations

ncRNA, non-coding RNA; sRNA, small RNA

## Authors' contributions

GG and IMA performed RNA analyses in the laboratory, BV designed and carried out bioinformatic analyses and participated in drafting the manuscript. WRH designed research and wrote the manuscript. All authors read and approved the final manuscript.

## Supplementary Material

Additional file 1 Results and conditions of an RNA motif-based search for Yfr1 homologs in *Prochlorococcus *sp. SS120 and MIT9211 with relaxed conditions. Click here for file
