# Supplementary material for: A motif-based search in bacterial genomes identifies the ortholog of the small RNA Yfr1 in all lineages of cyanobacteria
Source: BMC Genomics. 2007 Oct 17;8:375. doi: 10.1186/1471-2164-8-375 (PMC2190773; doi:10.1186/1471-2164-8-375)
Supplement: Additional file 1 — Results and conditions of an RNA motif-based search for Yfr1 homologs in Prochlorococcus sp. SS120 and MIT9211 with relaxed conditions. [file 1471-2164-8-375-S1.doc]

**Additional data - additional file 1**

**Search for Yfr1 homologs in *Prochlorococcus* sp. SS120 and MIT9211 with relaxed conditions:** Two mismatches were allowed in the central consensus element the required minimum score was reduced to 0.0, the search space contained the complete genome sequence, including annotated protein-coding genes.

RNA motif descriptor for the relaxed conditions

descr

h5( minlen=5, maxlen=10, mispair=1, pair+={"g:u","u:g"} ) ss( minlen=3, maxlen=8 ) h3

ss( minlen=12, maxlen=20, seq="ACTCCTCACAC", **mismatch<=2**)

h5( minlen=6, maxlen=9, mispair=0, pair+={"g:u","u:g"} )

ss( minlen=3, maxlen=15 ) h3

ss( seq="^[AUCG]UU$")

score

{ gcnt = 0;

len = length( h5[5] );

loop = length( ss[6] );

for( i = 1; i <= len; i++ ){ j = len - i + 1;

b1 = h5[5,i,1]; b2 = h3[7,j,1];

if( (b1 == "g" && b2 == "c") || (b1 == "c" && b2 == "g") ) gcnt++;

} # require 65% GC in the stem!

SCORE = 1.0 * gcnt / len; **if( SCORE < 0.00) REJECT;**

#RM scored

#RM descr h5 ss h3 ss h5 ss h3 ss

#RM dfile yfr1.rnamotif

**Candidate Yfr1 homologs in *Prochlorococcus* sp. SS120 under relaxed search conditions:**

**Genomic location 1**

>gi|33238865|gb|AE017126|AE017126 DEFINITION Prochlorococcus marinus subsp. marinus str. CCMP1375 complete genome.

gi|33238865|gb|AE017126|AE017126 score 0.000 0 start at base 284049 58 gttgaata gcctcat tattaaat actcctctcccaaaa atagtt tctgt aattgt ttt

**Genomic location 2**

>gi|33238865|gb|AE017126|AE017126 DEFINITION Prochlorococcus marinus subsp. marinus str. CCMP1375 complete genome.

gi|33238865|gb|AE017126|AE017126 score 0.000 0 start at base 331697 67 aatctg aacctttc cagttt gactccccagaccattcta ttttgaat ggagatatg atttgggg ctt

>gi|33238865|gb|AE017126|AE017126 DEFINITION Prochlorococcus marinus subsp. marinus str. CCMP1375 complete genome.

gi|33238865|gb|AE017126|AE017126 score 0.000 0 start at base 331701 63 tgaac ctttcca gtttg actccccagaccattcta ttttgaat ggagatatg atttgggg ctt

>gi|33238865|gb|AE017126|AE017126 DEFINITION Prochlorococcus marinus subsp. marinus str. CCMP1375 complete genome.

gi|33238865|gb|AE017126|AE017126 score 0.000 0 start at base 331703 61 aacct ttcc agttt gactccccagaccattcta ttttgaat ggagatatg atttgggg ctt

**Genomic location 3**

>gi|33238865|gb|AE017126|AE017126 DEFINITION Prochlorococcus marinus subsp. marinus str. CCMP1375 complete genome.

gi|33238865|gb|AE017126|AE017126 score 0.000 0 start at base 1003705 59 aagtttgt caaca acaaactt gctcctcacaatgcag tttttg aaggtca tgaaga att

**Genomic location 4**

>gi|33238865|gb|AE017126|AE017126 DEFINITION Prochlorococcus marinus subsp. marinus str. CCMP1375 complete genome.

gi|33238865|gb|AE017126|AE017126 score 0.000 0 start at base 1005768 63 gaactt gaatatgg aacttt aaactccttacaaaa taaatt aaagcaaaaagag gattta att

>gi|33238865|gb|AE017126|AE017126 DEFINITION Prochlorococcus marinus subsp. marinus str. CCMP1375 complete genome.

gi|33238865|gb|AE017126|AE017126 score 0.000 0 start at base 1005772 59 ttgaa tatggaac tttaa actccttacaaaa taaatt aaagcaaaaagag gattta att

**Genomic location 5**

>gi|33238865|gb|AE017126|AE017126 DEFINITION Prochlorococcus marinus subsp. marinus str. CCMP1375 complete genome.

gi|33238865|gb|AE017126|AE017126 score 0.111 0 start at base 1165070 73 atggt atgaacaa atttt ctcttcatactccacaaac tcgaaataa tatttgggaatggat ttattttga att

>gi|33238865|gb|AE017126|AE017126 DEFINITION Prochlorococcus marinus subsp. marinus str. CCMP1375 complete genome.

gi|33238865|gb|AE017126|AE017126 score 0.111 0 start at base 1165072 71 ggtat gaacaa atttt ctcttcatactccacaaac tcgaaataa tatttgggaatggat ttattttga att

>gi|33238865|gb|AE017126|AE017126 DEFINITION Prochlorococcus marinus subsp. marinus str. CCMP1375 complete genome.

gi|33238865|gb|AE017126|AE017126 score 0.111 0 start at base 1165075 68 atgaa caaat tttct cttcatactccacaaac tcgaaataa tatttgggaatggat ttattttga att

>gi|33238865|gb|AE017126|AE017126 DEFINITION Prochlorococcus marinus subsp. marinus str. CCMP1375 complete genome.

gi|33238865|gb|AE017126|AE017126 score 0.111 0 start at base 1165076 67 tgaaca aattttc tcttca tactccacaaac tcgaaataa tatttgggaatggat ttattttga att

**Genomic location 6**

>gi|33238865|gb|AE017126|AE017126 DEFINITION Prochlorococcus marinus subsp. marinus str. CCMP1375 complete genome.

gi|33238865|gb|AE017126|AE017126 score 0.000 1 start at base 1591426 59 tgctt taag aagaa atattcatcacaccatgg tttaaa aactttgtgaag tttagg att

>gi|33238865|gb|AE017126|AE017126 DEFINITION Prochlorococcus marinus subsp. marinus str. CCMP1375 complete genome.

gi|33238865|gb|AE017126|AE017126 score 0.000 1 start at base 1591425 58 gcttt aagaa gaaat attcatcacaccatgg tttaaa aactttgtgaag tttagg att

**Candidate Yfr1 homologs in *Prochlorococcus* sp. MIT 9211 under relaxed search conditions:**

**Genomic location 1**

>gi||gb|unknown|1099153590169 DEFINITION Prochlorococcus marinus str. MIT 9211.

gi||gb|unknown|1099153590169 score 0.143 0 start at base 567168 67 acaattg cgttttcc tcgttgt actcctcagaaatatgatta tctttat cgaaaggt atagagg att

>gi||gb|unknown|1099153590169 DEFINITION Prochlorococcus marinus str. MIT 9211.

gi||gb|unknown|1099153590169 score 0.143 0 start at base 567171 64 attgcg ttttcct cgttgt actcctcagaaatatgatta tctttat cgaaaggt atagagg att

>gi||gb|unknown|1099153590169 DEFINITION Prochlorococcus marinus str. MIT 9211.

gi||gb|unknown|1099153590169 score 0.143 0 start at base 567174 61 gcgtt ttcctc gttgt actcctcagaaatatgatta tctttat cgaaaggt atagagg att

**Genomic location 2**

>gi||gb|unknown|1099153590169 DEFINITION Prochlorococcus marinus str. MIT 9211.

gi||gb|unknown|1099153590169 score 0.667 0 start at base 627708 56 gatta ccactaac taaac aaaactcctcataaaaaaac ctccac ttt gtggag gtt

>gi||gb|unknown|1099153590169 DEFINITION Prochlorococcus marinus str. MIT 9211.

gi||gb|unknown|1099153590169 score 0.714 0 start at base 627708 57 gatta ccactaac taaac aaaactcctcataaaaaaa cctccac ttt gtggagg ttt

>gi||gb|unknown|1099153590169 DEFINITION Prochlorococcus marinus str. MIT 9211.

gi||gb|unknown|1099153590169 score 0.625 0 start at base 627708 58 gatta ccactaac taaac aaaactcctcataaaaaa acctccac ttt gtggaggt ttt

>gi||gb|unknown|1099153590169 DEFINITION Prochlorococcus marinus str. MIT 9211.

gi||gb|unknown|1099153590169 score 0.556 0 start at base 627708 59 gatta ccactaac taaac aaaactcctcataaaaa aacctccac ttt gtggaggtt ttt

>gi||gb|unknown|1099153590169 DEFINITION Prochlorococcus marinus str. MIT 9211.

gi||gb|unknown|1099153590169 score 0.444 0 start at base 627708 60 gatta ccactaac taaac aaaactcctcataaaa aaacctcca ctttg tggaggttt ttt

>gi||gb|unknown|1099153590169 DEFINITION Prochlorococcus marinus str. MIT 9211.

gi||gb|unknown|1099153590169 score 0.444 0 start at base 627708 61 gatta ccactaac taaac aaaactcctcataaa aaaacctcc actttgt ggaggtttt ttt

>gi||gb|unknown|1099153590169 DEFINITION Prochlorococcus marinus str. MIT 9211.

gi||gb|unknown|1099153590169 score 0.333 0 start at base 627708 62 gatta ccactaac taaac aaaactcctcataa aaaaacctc cactttgtg gaggttttt ttt

>gi||gb|unknown|1099153590169 DEFINITION Prochlorococcus marinus str. MIT 9211.

gi||gb|unknown|1099153590169 score 0.444 0 start at base 627708 60 gatta ccac taact aaacaaaactcctcataaaa aaacctcca ctttg tggaggttt ttt

>gi||gb|unknown|1099153590169 DEFINITION Prochlorococcus marinus str. MIT 9211.

gi||gb|unknown|1099153590169 score 0.444 0 start at base 627708 61 gatta ccac taact aaacaaaactcctcataaa aaaacctcc actttgt ggaggtttt ttt

>gi||gb|unknown|1099153590169 DEFINITION Prochlorococcus marinus str. MIT 9211.

gi||gb|unknown|1099153590169 score 0.333 0 start at base 627708 62 gatta ccac taact aaacaaaactcctcataa aaaaacctc cactttgtg gaggttttt ttt

**Genomic location 3**

>gi||gb|unknown|1099153590169 DEFINITION Prochlorococcus marinus str. MIT 9211.

gi||gb|unknown|1099153590169 score 0.222 0 start at base 694038 54 ttagc caaa gcaga actcattacacga tattggtcc cactta gggttggta ctt

>gi||gb|unknown|1099153590169 DEFINITION Prochlorococcus marinus str. MIT 9211.

gi||gb|unknown|1099153590169 score 0.667 0 start at base 967227 68 tgatta ccaaaag taatca actcctctcattcacaacaa aatgccccc ctcacaag gggggcatt ttt

>gi||gb|unknown|1099153590169 DEFINITION Prochlorococcus marinus str. MIT 9211.

gi||gb|unknown|1099153590169 score 0.667 0 start at base 967227 68 tgattac caaaa gtaatca actcctctcattcacaacaa aatgccccc ctcacaag gggggcatt ttt

>gi||gb|unknown|1099153590169 DEFINITION Prochlorococcus marinus str. MIT 9211.

gi||gb|unknown|1099153590169 score 0.556 0 start at base 967227 69 tgattac caaaa gtaatca actcctctcattcacaaca aaatgcccc cctcacaagg ggggcattt ttt

**Genomic location 4**

>gi||gb|unknown|1099153590169 DEFINITION Prochlorococcus marinus str. MIT 9211.

gi||gb|unknown|1099153590169 score 0.333 1 start at base 626367 59 tggaa aagcttca tttgg attcctcacactaaggac atccaa ttatagcc ttggat ctt

>gi||gb|unknown|1099153590169 DEFINITION Prochlorococcus marinus str. MIT 9211.

gi||gb|unknown|1099153590169 score 0.333 1 start at base 626364 56 aaaag ctt cattt ggattcctcacactaaggac atccaa ttatagcc ttggat ctt
